# Teachers' psychological resistance to digital innovation in jordanian entrepreneurship and business schools: Moderation of teachers' psychology and attitude toward educational technologies

**DOI:** 10.3389/fpsyg.2022.1004078

**Published:** 2022-09-26

**Authors:** Suhaib Khalid AL-Takhayneh, Wejdan Karaki, Rashad Ahmad Hasan, Bang-Lee Chang, Junaid M. Shaikh, Wajiha Kanwal

**Affiliations:** ^1^Department of Guidance and Special Education, Faculty of Educational Sciences, Mu'tah University, Kerak, Jordan; ^2^Department of Psychology, Faculty of Educational Sciences, Mu'tah University, Kerak, Jordan; ^3^Department of Counselling and Mental Health, The Ministry of Education, University Brigade/Capital Governorate, Amman, Jordan; ^4^Department of Architecture and Urban Design, College of Environmental Design, Chinese Culture University, Taipei, Taiwan; ^5^Department of Accounting, School of Business, University of Technology Brunei, Jerudong, Brunei; ^6^Department of Education, University of Wah, Wah Cantt, Pakistan

**Keywords:** school innovation climate, school culture, resistance to digital innovation, attitude toward technology, business school teachers, entrepreneurship

## Abstract

The current study aimed to highlight the factors that may influence teachers' psychological resistance to digital technologies in entrepreneurship and business schools. Theoretically grounded in the diffusion of innovations theory and the theory of planned behavior, the current research investigates teachers' psychological resistance to digital innovation, school culture and climate, and moderation of teacher attitudes toward educational technologies. A cross-sectional field survey of 600 business and entrepreneurship school teachers was conducted in Jordan. In this study, partial least square-structural equation modeling (PLS-SEM) was used to assess the variables' “direct and moderating impacts” using the Smart PLS software 3.0. According to the results, school culture and school innovation climate had a considerable positive impact on teachers' resistance to digital innovation. Additionally, teachers' attitudes toward educational technologies moderated the relationship between study constructs in the framework. The study is a significant advance to the literature related to entrepreneurship, business education, and digital innovation. Several key policy insights and recommendations for further research, as well as theoretical and practical implications, are suggested.

## Introduction

The concept and strategies for disseminating new technology are known as an innovation which is considered vital for transformation. Among the different components of transformation are change and creativity (Aguilar, 2018). The uniqueness is created to be more productive in attaining a competitive advantage for the organization with its distinctive features (Toto and Limone, 2021b). Teachers can be a strong factor for human transformation, economic motion, and social inclusion only if it serves anyone, or, as John Dewy puts it, “if it helps to the proper growth of all members of society” (Weintrobe, 1970). Resistance to innovation has been described as the resistance offered by end users to an innovation, “either because it poses potential changes from a satisfactory status quo or because it conflicts with their belief structure” (Abbas and Awan, 2017). The role of this psychological resistance is vital in determining the successful adoption of innovation because it has the potential to stop or delay the acceptance by end users. It has been considered among the top reasons for technology avoidance and hurdle in the successful implementation of technology (Toto and Limone, 2021a). Teachers' resistance to digital innovation has been and will continue to be a serious concern for educational institutions in the following decade (Håkansson, 2019). Business managers and entrepreneurs concerned with ensuring that digitalization is changing and the application of new technologies in the education sector must include teacher resistance as a subject of investigation (Ziauddin et al., 2010; Toto and Limone, 2021b; Ahmad, 2022). The current research is responding to this call for research and is incremental to respond to these indicated research gaps in innovation and entrepreneurship integration with education literature.

The climate of innovation at a business school is a constant aspect that sets it apart from other schools and educational settings (Fidan and Oztürk, 2015). It is made up of common views of basic characteristics including independence, confidence, cooperation, approbation, creativity, and fairness that are formed by the institutions' stakeholders' mutual relationship with each other (Allen et al., 2015; Ain et al., 2016; Zarb, 2016; Bint-e-Nasir et al., 2021; Gupta, 2022). The school innovation climate serves as a foundation for interpreting the instances that people of the institution endure, as an intermediary that makes the institution's prevalent morals, social rules, and preferences, and as a major influence that forms employees' behavior (Ockwell and Byrne, 2016). According to Newman et al. (2020), school innovation climate criteria can be classified into two parts: “(1) the cognitive schema approach, which considers school innovation climate to be cognitive descriptions created by individuals concerning their work environments, and (2) the shared perceptions approach, which considers school innovation climate to be members' shared perceptions of policies, applications, and operations” (Mathisen et al., 2006; Kuenzi and Schminke, 2009). Studies linking school innovation climate with teachers' resistance to digital technologies are scarce in the literature, and specially focused studies on entrepreneurship and business school teachers' innovation resistance are rare. Hence, the current study is among the earliest to theoretically establish and test this conceptualization for future growth in this stream of literature.

School culture can be defined as the spirit and social ambiance within the school including management and institutional structure about values and norms to be practiced by all members to achieve learning objectives (Schipper et al., 2020). According to previous studies, implementing new technology in schools necessitates the main challenge in school culture and entails a broad range of educational, technical, and administrative aspects, the complex relationships of which are not truly recognized (Little, 2012; Baricaua, 2016; Schipper et al., 2018). An isolated school culture, in which cooperative learning is not prevalent, severely inhibits strong educational experiences (Baricaua, 2016) and may result in personal difficulties (Avidov-Ungar and Eshet-Alkalai, 2011) and contributes to eroding trust and personality attitudes (Rozimela, 2020). However, altering the existing values and culture of the school and educational institutions into different cultures may be a big challenge (Schipper et al., 2020). It can be challenging to connect to cooperative research and learning, as well as topic emphasis and alignment with the school community and teachers' expertise and attitudes (Baricaua, 2016). This study is novel to advance in literature by theoretically bridging the research gap of linking school culture with teachers' resistance to digital innovation, especially in business and entrepreneurship school contexts.

Teachers' willingness to become full collaborators in the learning process for students and their attitudes toward educational technologies are critical success factors for determining students outcomes (Avidov-Ungar and Eshet-Alkalai, 2011; Yilmaz and Bayraktar, 2014; Çoklar and Özbek, 2017). Likewise, change is seen as one of the most common causes of transformation loss in businesses in general and educational institutions such as entrepreneurship schools (Jam et al., 2011; Waheed et al., 2016b; Dashtestani, 2020). Many researchers contend that instructors' opposition to advanced technology deployment in schools is the most crucial aspect in completing a project (Canals and Al-Rawashdeh, 2019), owing to the technology's incompatibility with their educational ideas and traditions (Donaghue, 2015; Dashtestani, 2020). Many researchers have identified, unearthed, and examined the basic elements that influence teachers' attitudes toward educational technologies, which include internal focus, teacher engagement in continuous improvement decisions, and a fear of change (Alhamami and Costello, 2019). This attitude may play a significant decisive role in determining teachers' resistance to digital innovation, especially in entrepreneurship schools. Thus, current research made an advance to literature by examining the moderating role of teachers' attitudes toward technology between school innovation climate, school culture, and teachers' resistance to digital innovation. Such research attempts are novel in terms of theoretical grounding and help the field grow for effective technology adoption in business and entrepreneurship schools.

Furthermore, the current research was conducted with teachers from higher education schools, especially focusing on entrepreneurship and business schools working under universities in Jordan. The study context of Jordan has several important and logical reasons to be considered an appropriate study setting for current research. Jordan is a small country with a struggling economy and scarce resources in the Middle East. The number of graduates in Jordan is among the top countries in the region as well as its rates of unemployment are higher than other Middle Eastern countries (Kayed et al., 2022). The continuous rise of migrants from Syria has resulted in a continuous increase in unemployment ratios from 18.7, 19.2, and 24.7% in the consecutive 3 years of 2018–2020. Besides immigrants, the COVID-19 crisis has also impacted this small country's higher unemployment level (Mugableh, 2021). The recent pressures on the Jordanian economy and society have highlighted the necessity of knowledge and skill transfers from universities to industrial outlets (Kayed et al., 2022). However, the concept of education related to entrepreneurship is still nascent in the country (Sandri, 2016). Some recent studies on entrepreneurial education in Jordan have recommended further studies in this domain, especially linking with technology and innovation (Abu-Rumman et al., 2022; Kayed et al., 2022). Recent studies identified a strong need in a similar context, and researchers were encouraged to identify the factors influencing innovativeness among Jordanian entrepreneurship and business students (Abu-Rumman et al., 2022). Hence providing a clear research gap to be filled and a significant contextual advance made by this research to study school innovation and technology adoption-related constructs in Jordanian teachers' sample and bring key insights from collectivist Jordanian culture.

Finally, the current study has been based on the diffusion of innovations theory and theory of planned behavior (TPB). Diffusion of innovation theory is described as “the successful integration of educational technology depends on the attitudes and aptitudes of teachers” (Hart and Laher, 2015). The planned behavior theory is determined by “attitude (e.g., technology innovation acceptance), subjective norm (e.g., the organization's innovation climate), and behavior control consciousness (e.g., the innovative teaching behavior with ICT subscale)” (Chou et al., 2019). The research deals with teachers' resistance to digital innovation, school culture, school innovation climate, and moderation of teachers' attitudes toward educational technologies. As a result, these ideas serve as a basis for the suggested theoretical framework being tested empirically in this study. The purpose of this research is to highlight the following research objectives to identify and achieve them:

- To examine the impact of school innovation climate on teachers' resistance to digital innovation.
- To examine the impact of school culture on teachers' resistance to digital innovation.
- To examine the moderating effect of the teachers' attitude toward educational technologies on the relationship between school innovation climate, school culture, and teachers' resistance to digital innovations.

## Literature review

The current research focuses on teachers' resistance to digital innovation, school culture and school innovation climate, and moderation of teachers' attitudes toward educational technologies between study constructs. The current study has been based on the diffusion of innovations theory and theory of planned behavior (TPB). Diffusion of innovation theory is described as “the successful integration of educational technology depends on the attitudes and aptitudes of teachers” (Hart and Laher, 2015). According to Min et al. (2019), perceptions at this stage are influenced by how beneficial a person interprets the technology in terms of its overall opportunity over data analysis techniques, integration with existing practices, difficulty, or whether the reforms introduced about through technology are visible and noticeable, allowing the idea to be tested before integration. The planned behavior theory is determined by “attitude (e.g., technology innovation acceptance), subjective norm (e.g., the organization's innovation climate), and behavior control consciousness (e.g., the innovative teaching behavior with ICT subscale)” (Chou et al., 2019). The climate and culture of a business and entrepreneurship school, such as cohesiveness, work flexibility, teamwork, and inventive attitude, enable instructors to utilize new and effective teaching strategies to keep students' interest, engage them to study, and generate better learning outcomes (Thurlings et al., 2015). The relevant institutional and research objectives described in the research are crucial in uniting diverse theories into a consistent framework related to teacher resistance to digital innovation, school culture, and climate, and moderation of teachers' attitude toward educational technologies is a unique theoretical combination to explain a scarcely researched scientific conceptualization. These two theories explain the behavior of individuals' resistance and their motives and psychological interests behind this resistance are influenced by their attitude toward technology, thus providing a strong theoretical base for the proposed conceptual model in this study.

### Business/entrepreneurship school innovation climate and teachers' resistance to digital innovation

According to Fidan and Oztürk (2015), the climate is a feature of organizations that is best described as a collection of ideas, sentiments, and behavior. The atmosphere sends messages about how things are done in the business and helps to maintain and promote a shared vision of reality among employees. Chang et al. (2011) explained that an institution's atmosphere influences job performance, inventiveness, work contentment, and profitability. According to Fischer and Riedl (2020), a sense of control can drive employees to generate new ideas in difficult and stressful work environments. According to encouraging invention nurtures innovation in educational institutions and business organizations. Moreover, according to Naseer et al. (2021), the school climate for innovation is influenced by supporting creativity, allocating assistance in the workplace, and applying innovative approaches. The institutional climate is a constant trait that distinguishes one organization from another (Ye et al., 2022). It comprises collective beliefs of concept characteristics including independence, confidence, cooperation, approbation, creativity, and fairness that are formed by the institution's people's positive correlations (Ockwell and Byrne, 2016). Climate for school serves as a foundation for interpreting the conditions that people of the institution confront, as an intermediary that makes the institution's prevalent beliefs, rules, and trends evident, and as a source of influence that molds people's behavior (Ye et al., 2022). A school innovation climate refers to “teachers' perceptions of their school encouraging innovative teaching behavior, creative thinking, and providing ICT teaching resources” (Nuchso et al., 2016; Waheed et al., 2016a; Turnheim et al., 2018; Fischer and Riedl, 2020; Pandi and Chinnasamy, 2022).

According to studies on educational technology, teachers' attitudes significantly impact the implementation of new technology in schools. Teachers' attitudes toward educational technology should be encouraging, and they should be taught to use it in the field of teaching to employ educational technology in business and entrepreneurship schools. During the learning experience, prospective instructors should be motivated to employ educational technologies in instructional applications (Ozyürek and Ulutürk, 2016; Meidrina et al., 2017; Turnheim et al., 2018; Fischer and Riedl, 2020). Teaching applications are important for teacher candidates for various reasons, including familiarization with their future schools and education climates, people skills with students, evaluation of educators' thinking levels, and academic semester of teacher–student relationships. In addition, recent studies indicate that teachers' attitudes toward educational technology have significant implications for their behaviors in using educational technology for teaching (Ye et al., 2022). Thus, an innovative climate is expected to inverse teachers' resistance to digital innovation. Hence, the following hypothesis is suggested;

H1: School innovation climate is significantly related to teachers' resistance to digital innovation.

### School culture and teachers' resistance to digital innovation

Hart and Laher (2015) noticed that schools had their own identity, with elaborated traditions of personal connections and a set of cultural and moral standards were the first to use the term culture to characterize life inside schools (Schipper et al., 2020). In the 1980s, the concept of school culture began to gain attraction in the research community (Effectiveness and Improvement; Schoen and Teddlie, 2008; Tezci, 2011; Kalkan et al., 2020) and termed a component associated with system effectiveness (Tezci, 2011; Ozgenel, 2020). Education experts may have paid less attention to culture because it is linked to an organization's assumed beliefs, underlying assumptions, expectations, collective memories, and definitions (Kalkan et al., 2020). Many scholars have looked at the impact of culture on organizational behavior, resulting in various descriptions, especially in business management and entrepreneurship domains. Some of these range from broad cultural understandings to a larger level, in which distinctions between different regions across the globe have been mentioned along with numerous aspects (Qazi et al., 2014; Abdullah, 2019; Moschogianni, 2022). Additionally, defined cultural impacts research has studied differences in the culture of social and cultural categories gender-based (Abdullah, 2019), race (Schipper et al., 2020), profession (Atasoy, 2020), and (Effectiveness and Improvement). Many other scholars have investigated the dominant culture within a certain institution (Schoen and Teddlie, 2008). This last aspect has been utilized to research schools to identify disparities in institutional school culture (Tezci, 2011; Abdullah, 2019).

In a school culture, where education is widely seen as a suitable replacement for the acquired status quo and where calls for equality and fairness have become louder, change is seen as a challenge to the leadership's entitlements and is thus fiercely fought. These forces are most active on a government level (Kalkan et al., 2020). However, resistance to change within the schooling institutions can be much stronger due to the system's administrators' more apparent special interests in the established order (Shamim, 2014). Teachers' resistance to innovation overlaps design and creativity, requiring the application, implementation, and explanation of new ideas to deliver an intended result, such as a new customer, new market, larger market, or competitive advantage” (Toto and Limone, 2021a). It is expected that school culture has great potential to become an effective determinant of teachers' resistance to digital innovation. Thus, based on the above in-depth literature support following hypothesis is suggested;

H2: School culture significantly impacts teachers' resistance to digital innovation.

### Moderating effect of the teachers' attitude toward educational technologies

Even teachers with prior skill and proven views about the use of technologies, according to Avidov-Ungar and Eshet-Alkalai (2011), may be distanced from this practice due to the institutional culture of the context in which they educate. In essence, they find that good attitudes are insufficient if additional hurdles exist, such as a lack of managerial support or time to prepare for technology implementation. School teachers will be less willing to welcome changes if they are only receivers of them instead of contributors to the judgment that led to them (Canals and Al-Rawashdeh, 2019). Furthermore, whether or not to accept specific technological solutions will be determined by their position within the institution and their ability to exercise both freedom and free expression (Hart and Laher, 2015). Yilmaz and Bayraktar (2014) also reported that a successful education system significantly affects attitudes toward teaching methods and that students' intentions, shared perceptions, and separate roles in the procedure are related to teachers' commitment to compromise.

Toto and Limone (2021a) investigated the origins of teacher resistance to digital learning, which included resistance to change. The extent of participation in the change effort, optimism for the change's potential combined with fears about its risks, and personal variables. Moreover, Ye et al. (2022) emphasize that teachers play a critical role in the change initiative, and their resistance highlights the need for frameworks that allow them to participate in that change, even though most online systems are still focused on leading choices, as the authors point out. Teachers' cognitive biases, such as the endowment effect and loss aversion, might be linked to patterns of faculty resistance to change (Sanni et al., 2013; Kalkan et al., 2020; Muhammad et al., 2021). Several other empirical research studies have examined the characteristics that influence the faculty adoption of innovation and the perceived barriers to technology use in language instruction schools (Avidov-Ungar and Eshet-Alkalai, 2011; Aguilar, 2018; Canals and Al-Rawashdeh, 2019; Chou et al., 2019). Some teachers are more hesitant to incorporate new technologies into their profession than others and it becomes more challenging in entrepreneurship and business schools. Understanding the elements that drive innovation adoption aids in the design and content of staff development programs that prepare educators to adopt technology into education (Shahbaz et al., 2014; Musa, 2016; Abbas and Awan, 2017). The scholars concluded that teachers often do not comprehend how their students use technology, and students do not know their teachers' intentions. Teacher resentment can be traced back to various sources (Avidov-Ungar and Eshet-Alkalai, 2011; Aguilar, 2018; Canals and Al-Rawashdeh, 2019; Chou et al., 2019). Thus, teachers' attitudes toward technology may influence the association between school culture, school innovation climate, and teachers' resistance to digital technology in entrepreneurship and business schools. Hence, the following hypotheses are suggested;

H3: Teachers' attitudes toward educational technologies moderate the relationship between school innovation climate, school culture, and teachers' resistance to digital innovations. A higher level of attitude toward technology adoption will help overcome the resistance to digital innovation among teachers.

## Research methodology

**Figure 1:**
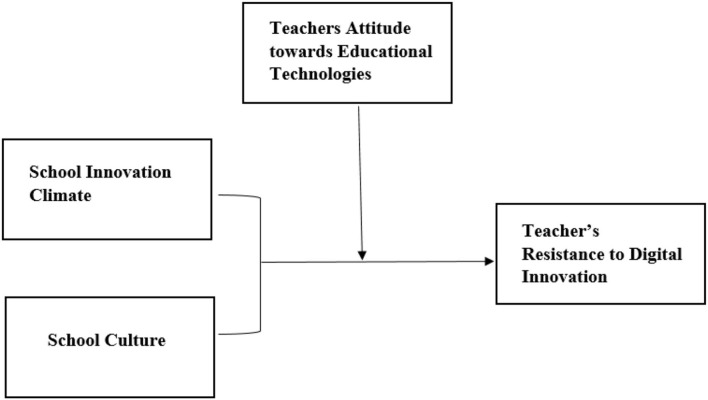
Theoretical framework development.

Based on a detailed assessment of the literature and the diffusion of innovations theory and theory of planned behavior, the conceptual framework in Figure 1 was established, and hypotheses were proposed for empirical testing. Multiple sources that contribute to the development of an entrepreneurial mentality are documented in several studies. According to Canals and Al-Rawashdeh (2019), the educational environment significantly influences a teacher's attitude toward digital innovation. Three crucial factors influencing attitudes are the inclination for trying new things, student engagement, and the desire for success. Ye et al. (2022) discovered that entrepreneurial school innovation climate, culture, and teachers' attitudes toward digital innovation boost profitability. Each nation has strived to adopt practical and consistent instructional innovations to address the demands (Toto and Limone, 2021a). The growth rate of entrepreneurial activities in Jordan is very low compared to developed nations such as China and the USA (Abu-Rumman et al., 2022). Contrarily, the opportunities for entrepreneurial activities and new start-ups are very high ((Monitor, 2019)). It is visible from the recent progress that World Economic Forum has included 27 ventures from Jordan in the list of top 100 ventures in Arabic countries (Kayed et al., 2022). The Jordanian government is trying hard to promote entrepreneurial activities in Jordan (Abukumail, 2019), but it still faces several significant challenges in promoting entrepreneurial activities and education related to creativity and innovation (Abu-Rumman et al., 2022). The recent developments in GCC countries have started a race toward technology and innovation adoption. Thus providing a huge research gap to study technological resistance among teachers in entrepreneurship and business schools in Jordan (Soto-Acosta et al., 2016). Recent studies reported a lack of innovativeness among Jordanian entrepreneurs and business professionals (Syed et al., 2020; Abu-Rumman et al., 2022). This has pointed toward an emerging research problem in which current study has attempted to address by focusing on factors affecting the psychological resistance of teachers in Jordanian business and entrepreneurial schools. It becomes an emerging research question to study why the highest number of the graduate-producing country is lacking in entrepreneurship contribution among GCC nations. Thus, pointing toward some adaptability issues in the teaching system of business schools and entrepreneurship schools. In particular, technology adoption has been an emerging area of concern in educational institutions. This situation makes teachers in entrepreneurship and business schools the right population for sampling choice in this study. The current research adopted a selective approach with a convenience sampling technique that adequately matched with objectives of the study. Only the teachers in entrepreneurship and business schools were selected, and the data were collected based on their willingness and time availability. This type of sampling technique in previous literature has been objected to by researchers (Etikan et al., 2016). However, despite criticism still, convenience sampling has been used in various studies frequently (Jager et al., 2017; Kempen and Tobias-Mamina, 2022). The current study examines teachers' resistance to digital innovation in entrepreneurship and business schools in Jordan. Thus, the convenience sampling approach was an appropriate technique for this current study.

The sample is a proper representative of a study population which has proper attributes the same as the study population (Majid, 2018). According to Hair et al. (2014), the following criteria were used to establish the sample size for structural equation modeling analysis.


$$\begin{array}{l} \text{Sample size}=(\text{Number of indicators}\\ \;+\text{Number of latent variables})\\ \;\times (\text{Estimated}\;\text{Parameters})\\ (22+4)\times (8)=208 \end{array}$$

As it is a minimum defined sample size but it is always advised to select a sample two times or three times bigger than the minimum sample limit (Hair et al., 2014). Hence, the current study decided to target three times the sample of the targeted population 208 × 3 = 624, to be on the safer side, 700 teachers were targeted to be included in the study, and finally, this study concluded with a final response rate of 600 respondents.

### Participants and procedure

A letter explaining the study's aims and objectives was drafted and delivered to the administrations of business and entrepreneurship schools in Jordan to acquire formal approval to involve their faculty in this research investigation. School administrators were ensured for strict anonymity that neither their schools nor their universities will be identified and the identifiable demographic data will never be shared with any third party at any stage of this research project. Some schools refused to participate for policy reasons, while five business and entrepreneurship schools agreed to participate in this study. After approval, the researchers visited the universities and business schools and approached the faculty members and teachers to seek their voluntary participation in this study. They were also ensured for anonymity and confidentiality of their responses, and only aggregate results will be used for publishing purposes where neither individuals nor institutions will be identifiable for specific responses. A total of 700 faculty members were surveyed, and a covering message detailing the research's goals and asking for their willingness to participate was sent along with the survey. As English is a widely used academic language in Jordanian business schools, all faculty members were experts to understand the survey language English. Some of the agreed participants were either uncomfortable or unavailable at the time of the survey. So finally, the researchers were able to enlist a total of 650 teachers who willingly and voluntarily participated in the study. The data gathering process began on 1 March 2022, and 600 completed questionnaires were collected by 20 April 2022. As a result, the ultimate response rate of 85% was retained by this study.

### Measures of the study

- A 22-item questionnaire was devised to analyze the teachers' resistance to digital innovation, school culture and school innovation climate, and moderation of teachers' attitudes toward educational technologies in teachers of business and entrepreneurship schools in Jordan.
- A four-item scale of school innovation climate was adopted (Remneland-Wikhamn and Wikhamn, 2011). Items included in this scale are “Our institution provides time and resources for teachers to generate, share/exchange, and experiment with innovative ideas/solutions” and “Our teachers are recognized and rewarded for their creativity and innovative ideas.” The responses were collected by a “7-point Likert scale ranging from 1= strongly disagree to 7 = strongly agree”.
- A five-item scale of school culture was adopted by Hart and Laher (2015). Items included in this scale are “We need computers and other educational technology that better suit the Jordanian culture and identity” and “Computers and other educational technology are proliferating too fast.” The responses were collected by a “7-point Likert scale ranging from 1= strongly disagree to 7 = strongly agree”.
- An eight-item scale of teachers' attitudes toward educational technologies was adopted (Hart and Laher, 2015). Items included in this scale are “I am glad there are more computers and other educational technology these days” and “Computers and other educational technology do more harm than good.” The responses were collected by a “7-point Likert scale ranging from 1= strongly disagree to 7 = strongly agree”.
- A five-item scale of teachers' resistance to digital innovation was adopted by Hosseini et al. (2016). Items included in this scale are “I fear of wasting my time using digital technologies” and “Resistance to change I fear of certain changes digital innovation may impose on me.” The responses were collected by a “7-point Likert scale ranging from 1= strongly disagree to 7 = strongly agree”.

## Data analysis

### Measurement model

The measurement and structural models were evaluated using SmartPLS3. According to the model assessment as shown in Table 1, respondents' gender and marital status significantly impacted their attitude and resistance to digital innovation among teachers. Hence, both demographic variables were controlled throughout the analysis.

**Table 1:** Demographic profile.

**Demography**	**Description**	**No. of Responses**	**%**
Gender	Male	270	45
	Female	330	55
Age	25–35	370	62
	Above 35	230	38
Qualification	Bachelors	350	58
	Master	250	42

Furthermore, as presented in Table 2, “Cronbach's (CA)” and “composite reliability (CR)” used the measurement model to assess the coherence of the measurements (Raeder et al., 2008). All investigation items had “CA and CR values larger than 0.7,” indicating that they met the reliability criterion (Ramayah et al., 2018). After that, “factor loadings” and “Average Variance Extracted” (AVE) were calculated to determine the constructs' convergent validity (Ramayah et al., 2018). In both studies, all factor loading of the research constructs exceeded the minimal criteria of 0.70, and AVE was greater than 0.50 (Raeder et al., 2008).

**Table 2:** Composite reliability, Cronbach's alpha and AVE values.

**Constructs/Items**	**CA**	**Rho-A**	**CR**	**AVE**
School culture	0.836	0.829	0.877	0.582
School innovation climate	0.805	0.814	0.873	0.633
Teachers attitude toward educational technologies	0.847	0.860	0.884	0.522
Teacher's resistance to digital innovation	0.868	0.881	0.904	0.654

CR, composite reliability; AVE, average variance extracted; CA, Cronbach's Alpha.

Moreover, as shown in Table 3, all study methods' discriminant validity must be proven. Fornell and Larcker (1981) described discriminant validity as “the extent to which a particular latent variable differs from other latent variables.” It was calculated by looking at the correlation between the analysis of variance items and the exact number of AVE (Raeder et al., 2008). Raeder et al. (2008) recommended that latent variables with a value of “0.50 or above” be employed to prove discriminant validity.

**Table 3:** Discriminant validity.

	**SC**	**SIC**	**TAET**	**TRDI**
School culture	0.695			
School innovation climate	0.457	0.796		
Teachers attitude toward educational technologies	0.486	0.333	0.723	
Teacher's resistance to digital innovation	0.797	0.585	0.566	0.809

SC, School culture; SIC, school innovation climate; TAET, teachers attitude toward educational technologies; TRDI, teachers' resistance to digital innovation.

### Assessment of structural model

As expressed in Figure 3. This part refers to the structural model expressed in evident measurement model connections (Raeder et al., 2008). The proposed model for the study uses a structural model to highlight the interconnectedness of the links.

**Figure 2:**
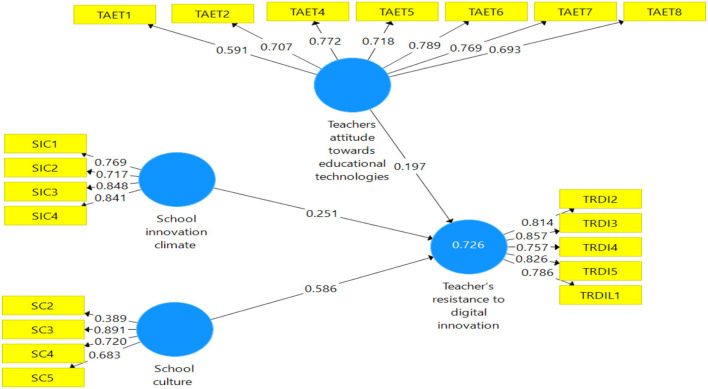
Assessment of PLS algorithm.

The structural model in PLS looks at the direct relationship between the offered hypotheses and their t-values and regression coefficients. An indirect effect is the same as a standardized beta value in regression analysis, according to Ramayah et al. (2018). The t-values and beta values of the regression coefficients are used to determine significance. According to Hair et al. (2017), t-values of more than “1.64” are statistically significant and are then used to make conclusions about the suggested hypothesis. The models' two main purposes are to examine direct linkages and to verify projected interactions between components using a structural model as presented in Figure 2.

**Figure 3:**
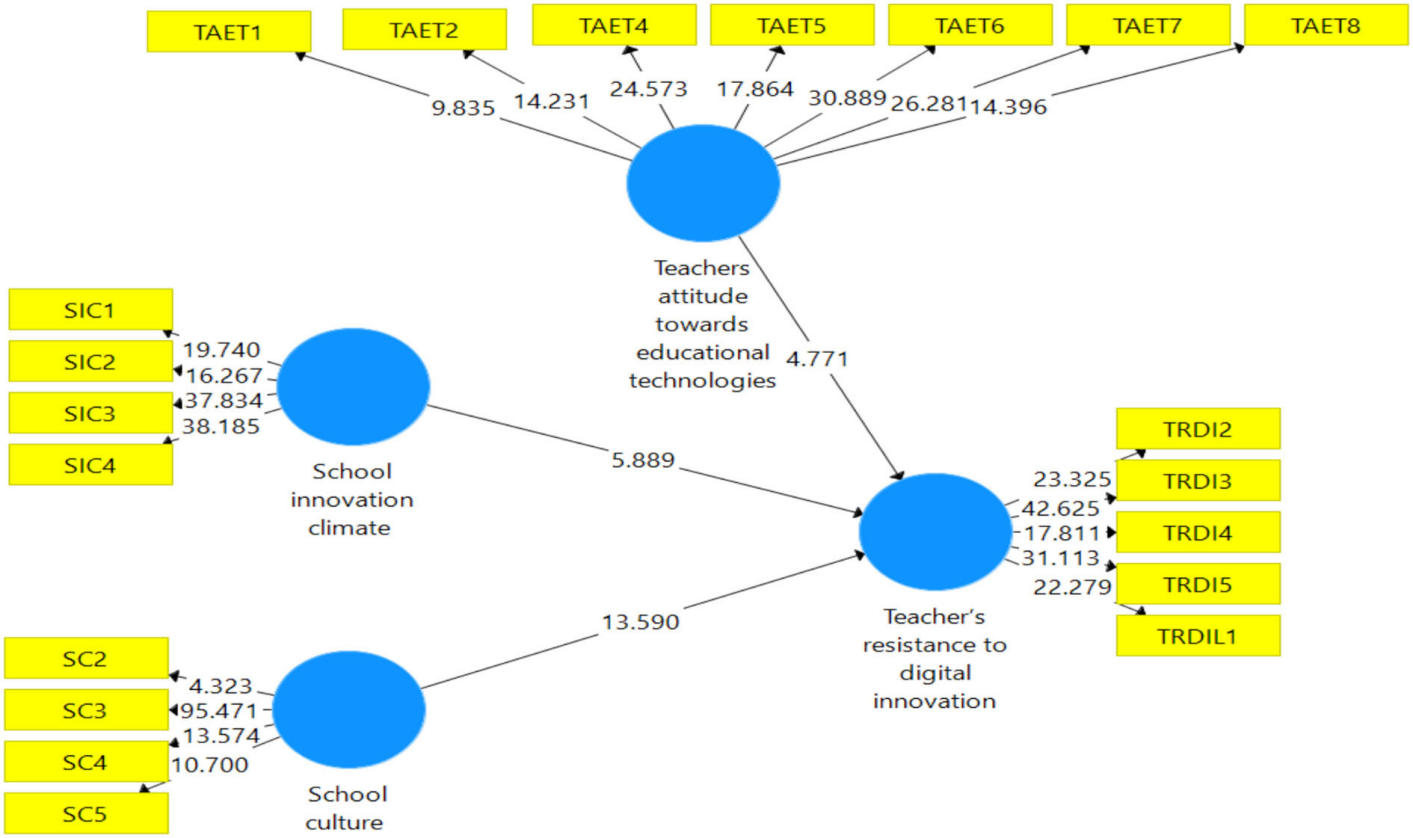
Assessment of PLS bootstraping.

Table 4 shows that the first hypothesis about school innovation climate's impact on teachers' resistance to digital innovation (B = 0.251, *p* < 0.000) was accepted. The second hypothesis was that school culture impact teachers' resistance to digital innovation (B = 0.586, *p* < 0.000), so this hypothesis was also accepted.

**Table 4:** Hypothesis testing.

**Path**	**B-value**	**Sample mean**	**Standard deviation**	***T* value**	***P*-value**	
SC -> TRDI	0.586	0.590	0.043	13.590	**0.000**	Supported
SIC -> TRDI	0.251	0.248	0.043	5.889	**0.000**	Supported

SC, School culture; SIC, school innovation climate; TRDI, teachers' resistance to digital innovation.

Table 5 shows that the third hypothesis of teachers' attitudes toward educational technologies (B = 0.77, *p* < 0.001) moderates the relationship between school culture and teachers' resistance toward digital innovation, so this part of the hypothesis was accepted. The second part of the hypothesis shows that teachers' attitude toward educational technologies (B = 0.083, *p* < 0.003) moderates the relationship between school innovation climate and teachers' resistance to digital innovations, so this hypothesis was also accepted.

**Table 5:** Moderator hypothesis testing.

**Path**	**B-value**	**(STDEV)**	***T*-value**	***P* value**	**Decision**
TRDI x TAET -> SC	0.077	0.056	4.234	0.001	Supported
TRDI x TAET -> SIC	0.083	0.047	3.476	0.003	Supported

SC, School culture; SIC, school innovation climate; TAET, teachers' attitude toward educational technologies; TRDI, teachers' resistance to digital innovation.

The value of *R*^2^ ranges from 0 to 1. Moreover, China (1998) recommended that the *R*^2^ of “0.13 is considered weak,” “0.33 is moderate,” and “0.67 is considered as strong.” The coefficient of determination for endogenous constructs is given in Table 6.

**Table 6:** Assessment of R square.

	**R^2^**
Teachers attitude toward educational technologies	0.726

## Discussion

The research objective of this study was to investigate the associations between school culture, school innovation climate, and teachers' resistance to digital innovation. Furthermore, the moderating impact of teachers' attitudes toward educational technology in the relationship between school culture, school innovation climate, and teachers' resistance to digital innovations were investigated. All of the hypotheses were supported by the findings.

The current study results depict that teachers are less resistant to digital innovations if school culture and school innovation climate are conducive to innovation emergence and that teachers' attitudes toward educational technologies are the determinants of this resistance toward technology innovation and change. Because the school innovation climate facilitates digital innovation adoption by teachers to help in overcoming the resistance to change. The first hypothesis of school innovation climate impact on teachers' resistance to digital innovation (B = 0.251, *p* < 0.000) gained significant support from study results, so this hypothesis was accepted. These results were indirectly consistent with the findings of Turnheim et al. (2018) and Fischer and Riedl (2020).

The second hypothesis shows that school culture's impact on teachers' resistance to digital innovation (B = 0.586, *p* < 0.000) was significant positively, so this hypothesis was also accepted. The second hypothesis's results were in line with the findings of previous studies (Tezci, 2011; Abdullah, 2019). The last and third hypotheses show that teachers' attitudes toward educational technologies moderated the relationship between school innovation climate and teachers' resistance to digital innovation (B = 0.083, *p* < 0.003). Moreover, teachers' attitudes toward technology also moderated between school culture and teachers' resistance to digital innovation (B = 0.77, *p* < 0.001), significant associations proved the moderation hypotheses. Although these hypotheses were proposed only in the current study, the results are consistent with theoretically supporting literature in the past. Several other empirical research studies have examined the characteristics that influence the faculty adoption of innovation and the perceived barriers to technology use in language instruction schools (Avidov-Ungar and Eshet-Alkalai, 2011; Aguilar, 2018; Canals and Al-Rawashdeh, 2019; Chou et al., 2019). Teacher resentment can be traced back to various sources (Hart and Laher, 2015). All these studies are in line with the current moderation findings significantly proved in this study. Conclusively, by managing attitude toward technology the resistance to digital innovations can be managed effectively and efficiently. However, resistance to change within the schooling institutions can be much stronger due to the system's administrators' more apparent special interests in the status quo.

### Theoretical implications

Theoretically, this article contributes to school culture and innovation climate, as well as teacher resistance to digital advancements. Thus, the current research was incremental to make numerous theoretical contributions to digital innovation and education management. To begin, this study is among the earliest studies on school climate, highlighting the importance of climate in building an environment conducive to institutional open innovation techniques and facilitating their adoption. Second, the goal was to investigate key components of the school innovation climate and to provide room for further scholarly debate on how to assess them in a transformation process incorporating school innovation climate in practice. Third, the findings are an excellent starting point for internal debates on whether the school innovation climate may be more linked with institutional theories and empirical studies. Lastly, the main objective of the study is to map the linkages between entrepreneurship and business schools. Which provided a future direction for linking business and entrepreneurship theories with school climate and school culture. Even these theories may be integrated with technology adoption and technology resistance theories and employees' attitudes toward technology. Finally, this research contributed to unifying two diverse theories of diffusion of innovations theory and theory of planned behavior into a particular framework. As a result of this collaboration, new avenues for teacher resistance research with theoretical assumptions have emerged for future scholars. The study advances the theories of digital innovation, resistance to innovation, technological attitude, and acceptance research with school culture and innovation climate. Application of all these theories in entrepreneurship and business schools setting of developing gulf country context is another major theoretical advance by current research. The current research attempted to bridge the theoretical gap between various emerging fields of management, entrepreneurship, technology, innovation, and change management. Integrating together all these fields in a single and unique conceptual framework is a major advance pitched by current research. It will help further theoretical integration between various fields and will open new avenues of exploration for future scholars.

### Practical implications

Furthermore, this research provides policymakers, practitioners, and managers with relevant information in various ways. To begin, the current study shows that in Jordan, the school culture in the local cultural context is one of the most important factors in expanding the idea of teachers' resistance to digital innovation and determining the effectiveness of school innovation. This finding is consistent with previous research that the entrepreneurial focus and technological change adoption process is low in Jordan (Kayed et al., 2022). As a result, when managing teacher resistance, administrators, education leaders, and policymakers should search for criteria based on local and national culture frames to integrate school culture and school innovation climate for better outcomes. In conclusion, the researcher may assert a link between teachers' resistance to digital innovation and their attitudes toward educational technology. Modifying teachers' attitudes toward technology can manage the resistance to digital innovation. Thus, before implementing any emerging technologies, teachers' attitudes should be given prime importance for better acceptance and successful management of technology adoption in business and entrepreneurship schools. Organizations may borrow the idea from this research's findings to manage employees' technology attitudes for better results. As there is a high number of graduates among Gulf nations available in Jordan (Kayed et al., 2022), still this nation is far behind in entrepreneurship success as compared to UAE and other developing countries. This research also brings key policy insights for entrepreneurs and business leaders, and human resource professionals to pay special attention to managing employees' attitudes toward technology when hiring new employees or managing existing employees along with organizational culture and innovation climate within an organization. In the context of Jordan, the government may increase focus and resources toward entrepreneurship and business schools' teachers' training and development for obtaining good support in technology adoption. It will ultimately help in fostering an entrepreneurship attitude among Jordanian students. These recommendations may also be borrowed for other regions and developing countries settings.

### Contextual implications

The current study along with theoretical and practical implications also made a significant contextual advance to the body of knowledge. First of all, such studies in GCC and Jordanian contexts are very scarce that attempt to tap resistance to digital innovation among business and entrepreneurship teachers sample. Thus, providing fresh empirical evidence from such a cultural context that has higher levels of power distance and collectivist attributes is a significant contribution to literature. As Jordan has been listed as an emerging entrepreneurship potential country by (Global Entrepreneurship (Monitor, 2019)), so the current study is vital because this unique empirical evidence can be generalized to GCC nations. Thus, presenting and highlighting the importance of the current study in the regional context makes this research more significant for future studies in this region. Jordan has the most number of graduates produced among GCC nations (Abu-Rumman et al., 2022), so the current study is expected to provide fresh entrepreneurial insights for policymakers to utilize this enormous potential of the country and young graduates to provide structural support which can help to flourish entrepreneurial activities in the country.

### Limitations and future studies

The current study, like all other studies, contains substantial shortcomings that must be addressed in future research attempts related to these subject areas. Business and entrepreneurship school teachers in Jordan participated in the current study. As a result, extrapolating study findings to other industries and organizational contexts may be difficult because of cultural differences between schools and corporate organizations. Future research could encompass diverse digital innovation samples from industry and academia to bring better insights. Second, the data were collected in a cross-sectional format, despite the likelihood that future researchers may use a longitudinal study design for better causation. To generate more meaningful results in future studies, researchers should investigate variables that may additionally moderate or mediate the effects associations in the framework of this research. The current study utilized data from business school teachers; however, future studies may consider taking fresh empirical evidence from alumni of business and entrepreneurship schools who are engaged in practical entrepreneurship activities and link it to teachers' resistance to digital innovation. Finally, in future studies, a researcher can use national culture's influence on school culture and innovation climate for a more holistic point of view. Comparative studies among GCC countries and regional comparisons are recommended in future investigations to tap cultural variations among study findings.

## Conclusion

The study findings revealed that business and entrepreneurship teachers have a favorable attitude toward educational technology in Jordanian business schools, and it moderated the influence of school culture and school innovation climate associations with resistance to digital technologies. Human resource development sections in the education sector should aim to prepare teachers to be competent in using emerging innovations in educational technologies for better and more impactful learning. In particular, such capacity-building training and workshops are vital for schools providing entrepreneurship and business education in this digital era. Different instructional techniques for various departments could be presented in the framework of the resource production course, with instances of how they can be used. Teachers' resistance to digital innovation, school culture, and school innovation climate for teachers in Jordan bring empirical findings from a unique cultural context of the GCC nation, attracting increased attention from academics, practitioners, and researchers in the field. This study is incremental to help institutional leaders believe that teacher resistance can be managed by the management of attitudes toward adopting digital innovation in organizational and business school settings. The research establishes a solid foundation for policy development and future research avenues in teacher resistance to digital innovation, school culture, school innovation climate, and teacher attitudes for various theoretical and practical insights. This study made several theoretical, practical, and contextual advances to the body of knowledge, especially related to entrepreneurship education.

## Data availability statement

The raw data supporting the conclusions of this article will be made available by the authors, without undue reservation.

## Author contributions

SA-T contributed in idea development, worked and coordinated throughout the process. WK and RH helped in data collection and analysis as well as interpretation of results. B-LC has contributed in discussion write up and proof editing the manuscript. JS helped in theory building, and introduction writeup. WK helped to design and handle the method section as well as literature review. All authors contributed to the article and approved the submitted version.

## Conflict of interest

The authors declare that the research was conducted in the absence of any commercial or financial relationships that could be construed as a potential conflict of interest.

## Publisher's note

All claims expressed in this article are solely those of the authors and do not necessarily represent those of their affiliated organizations, or those of the publisher, the editors and the reviewers. Any product that may be evaluated in this article, or claim that may be made by its manufacturer, is not guaranteed or endorsed by the publisher.

## Figures and Tables

**Table:** Questionnaire items.

**Variables**	**Items**	**Sources**
School Innovation climate	1. Our institution provides time and resources for teachers to generate, share/exchange, and experiment with innovative ideas/solutions. 2. Our teachers are working in diversely skilled work groups where there is free and open communication among the group members. 3. Our teachers frequently encounter non-routine and challenging work that stimulates creativity. 4. Our teachers are recognized and rewarded for their creativity and innovative ideas.	Remneland-Wikhamn and Wikhamn, 2011
School culture	1. We need computers and other Educational Technology that better suit the South Kalimantan Indonesia culture and identity. 2. There are other social issues that need to be addressed before implementing computers and other Educational Technology in education. 3. Computers and other Educational Technology dehumanize society. 4. Working with computers and other Educational Technology does not diminish people's relationships with one another. 5. Computers and other Educational Technology encourage unethical practices.	Hart and Laher, 2015
Teachers attitude toward educational technologies	1. I am glad there are more computers and other Educational Technology these days. 2. I do not like talking with others about computers and other Educational Technology. 3. Using computers and other Educational Technology is enjoyable. 4. Students must use computers and other Educational Technology in all subject matter. 5. Computers and other Educational Technology would motivate students to do more studying. 6. Computers and other Educational Technology are a fast and efficient means of getting information. 7. Computers and other Educational Technology can enhance students' learning. 8. Computers and other Educational Technology do more harm than good.	Hart and Laher, 2015
Teacher's resistance to digital innovation	1. I need to clarify some queries and justify the reasons. 2. I am waiting for the right time and required capability to new innovation. 3. I fear of wasting my time using digital technologies. 4. I need to get a solution for some of my complaints and objections. 5. Resistance to change I fear of certain changes digital innovation may impose on me.	Hosseini et al., 2016
